# Review of the species of *Leptomias* Faust from Sichuan, China (Coleoptera, Curculionidae, Entiminae)

**DOI:** 10.3897/zookeys.678.12543

**Published:** 2017-06-07

**Authors:** Zhenzhen Song, Li Ren, Runzhi Zhang, Chenggang Zhou

**Affiliations:** 1 College of Plant Protection, Shandong Agricultural University, Taian 271000, China; 2 Key Laboratory of Zoological Systematics and Evolution, Institute of Zoology, Chinese Academy of Sciences, No. 1 Beichen West Road, Chaoyang District, Beijing 100101, China

**Keywords:** Distribution, *Geotragus*, new species, Tanymecini, Weevils

## Abstract

An account is given of the twelve species of *Leptomias* Faust, 1886 occurring in the Sichuan Province of China, including the description of a new species, *Leptomias verticalis* Ren, Zhang & Song, **sp. n.** from Jiulong County, Southwest Sichuan. New locality data and remarks for the other eleven species, a key to and distribution map of all twelve Sichuan species are provided. *Leptomias chenae* Alonso-Zarazaga & Ren is transferred to *Geotragus* Schoenherr, 1845, where its valid name is *G. granulatus* (Chao, 1980), **comb. n.** in application of Art. 59.4. Structural details of *Leptomias verticalis* and *Geotragus granulatus* are illustrated.

## Introduction

*Leptomias* Faust, 1886 is a diverse genus of flightless weevils (Coleoptera, Curculionidae) in the subfamily Entiminae, with a centre of distribution in China, India, Nepal, Afghanistan and Myanmar. It differs from related genera by having (i) the metanepisternum completely separated from the metaventrite and (ii) the upper edge of the scrobes directed towards the lower margin of the eye (Chao 1980).

Ninety per cent of the *Leptomias* species known from China occur in Xizang, Yunnan, Sichuan and Qinghai. During identification of specimens collected in Sichuan province, one new and one misplaced species were found. *Leptomias chenae* Alonso-Zarazaga & Ren, 2013 (renamed because of secondary homonymy of its original name, *Leptomias granulatus* Chao, 1980) does not belong in *Leptomias* but instead to the genus *Geotragus* Schoenherr, 1845. With the transfer of this species out of *Leptomias* effected and the new species described in this paper, the number of current species of *Leptomias* remains at 159 and that of species recorded from China at 89. The new species and the new combination are here documented and illustrated, along with a key to the species present in Sichuan. Moreover, species documented in Sichuan are usually described in Chinese and it might be difficult for most people to obtain information of their distribution.

## Material and methods

All specimens, including types, examined for this study are located in the following collections: Institute of Zoology, Chinese Academy of Sciences, Beijing, China (IZCAS); Forschungsmuseum Alexander Koenig, Bonn, Germany; Natural History Museum, London, UK; Naturkundemuseum, Berlin, Germany; Senckenberg Naturforschendes Museum, Frankfurt am Main, Germany; Senckenberg Naturhistorische Sammlungen, Dresden, Germany. The types of the new species are deposited in IZCAS.

Specimens were dissected after soaking them in soapy water overnight, for cleaning and softening, and the dissected parts were placed in a cold 10 % NaOH solution for 20 hours to macerate the soft tissues. After dissection, all parts were photographed and stored in glycerine in microvials pinned beneath the specimen from which they were dissected.

The morphological terminology used in this study mainly follows Ren (2013). Measurements were made using an ocular micrometre as follows: standard length – in dorsal view from anterior margin of thorax to apex of elytra along midline; pronotal length – in dorsal view from anterior margin to base along midline; pronotal width – in dorsal view across widest part; elytral length – in dorsal view along suture of elytra from base to apex; elytral width – in dorsal view across widest part; rostral length – in lateral view in a straight line from apex to anterior margin of eye; rostral width – in dorsal view across base of rostrum. Measurements are made in millimetres.

All observations and dissections were performed using a Nikon SMZ1500 stereo microscope. The habitus photographs were taken with a MP-E 65 macro lens mounted on a CANON EOS700D digital camera. Other photographs were taken with a CCD Qimagine MicroPublisher 5.0 RTV camera mounted on a Zeiss SteREO Discovery V.12 microscope. Extended-focus images were generated with Auto-Montage Pro 5.03.0061 and edited with Adobe Photoshop CS 14.0 if required.

Label data are given *verbatim*, with pinyin romanisation and comments in square brackets if labels are in Chinese; labels are separated by semicolons and lines by slashes.

## Taxonomic treatment

### Leptomias verticalis

Taxon classificationAnimaliaColeopteraCurculionidae

Ren, Zhang & Song sp. n.

http://zoobank.org/02A25235-8C44-4C3D-9270-AAD7BB9E9EC6

Figs 1–9 , 10–23

#### Diagnosis.

This new species resembles *L. ochrolineatus* Chen, 1987 but differs by the following characters: elytra in lateral view abruptly sloping posteriorly, dorsal edge of slope of declivity straight, almost parallel to anterior margin; elytra in dorsal view at apical 1/3 with symmetrical crescent-shaped dark brown patches.

#### Description.

Holotype, male. *Measurements* (mm): Standard length: 8.00; pronotal length: 3.00; pronotal width: 3.00; elytral length: 5.00; elytral width: 3.20; rostral length: 1.30; rostral width: 1.20.

**Figures 1–9.**
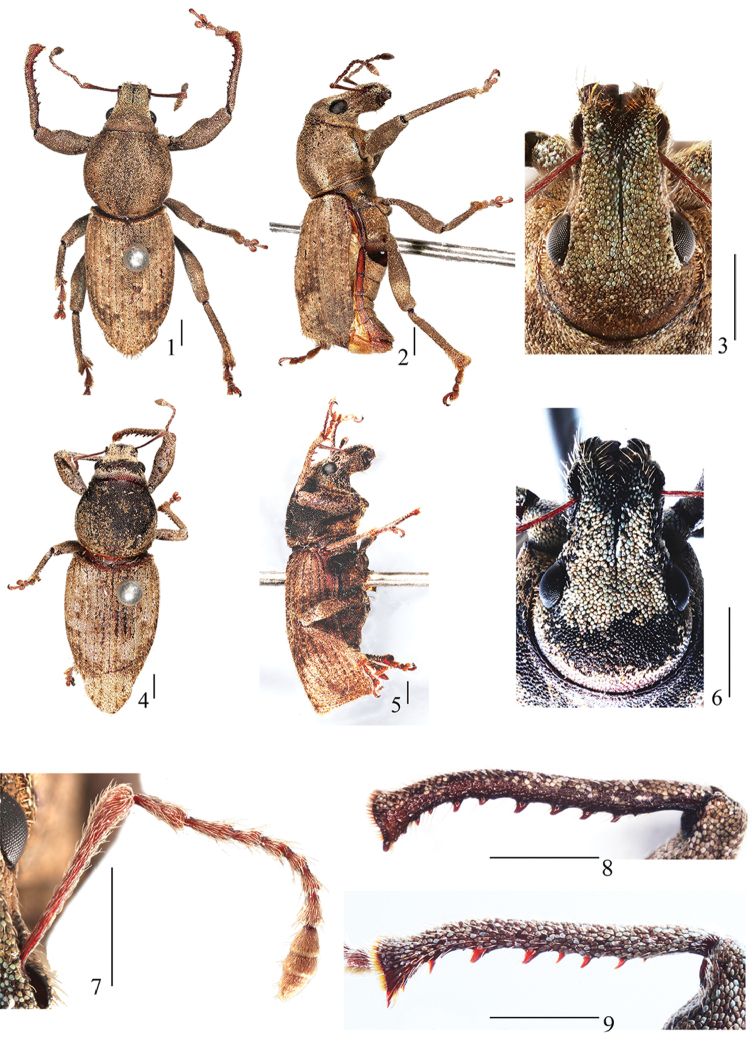
Habitus of *Leptomias verticalis* sp. n.: **1** male paratype, dorsal view **2** male paratype, lateral view **3** male paratype, head and rostrum, anterior view **4** female paratype, dorsal view **5** female paratype, lateral view **6** female paratype, head and rostrum, anterior view **7** male paratype, antenna, anterior view **8** male paratype, right protibia, anterior view **9** female paratype, right protibia, anterior view. Scale bars 1 mm.

*Habitus and colour* (Figs 1, 2): Body elongate-oval; integument dark reddish brown, antennae and legs reddish brown; with coppery to white to pale brown to brown scales, dorsal side of rostrum and apex of femora and tibiae with metallic turquoise scales; scales moderately dense, tessellate, contiguous but not overlapping, on dorsal side of rostrum moderately dense, oval to polygonal, behind epistome sparse, clearly different in colour from others, on lateral and ventral surfaces of rostrum moderately dense, around eyes penniform, elongate-oval, absent from anterior part of antennal scrobes but dense, penniform along posterior part, on pronotum dense, round to oval to polygonal, on elytra polygonal, moderately dense, at apical 1/3 forming symmetrical crescent-shaped dark brown patches, on ventrites dense, penniform, on legs dense, round to elongate-oval; body sparsely covered with recumbent to suberect, short and slightly fine, lanceolate setae, setae on rostrum subrecumbent, fine, lanceolate, sparse, on scapes and desmomeres 1–7 long, recumbent, fine, dense, on pronotum subrecumbent to erect, curved, on interstriae subrecumbent to erect, moderately thick, sparse, on tibiae long, moderately dense, lanceolate, on femora recumbent, short, fine, moderately dense.

*Head* (Fig. 3): Moderately convex; with small, sparse punctures and dense, tiny granules, each granule covered by a scale; eyes convex, moderately oval, with deep and fine circumocular sulcus along dorsal and anterior edge; between eyes moderately convex, higher than dorsal surface of rostrum in lateral view.

*Rostrum* (Fig. 3): In dorsal view 1.13× longer than wide, apex narrower than base; base slightly narrower than distance at midpoint between eyes; dorsal surface with narrow and deep median sulcus extending from posterior margin of frons to midpoint between eyes; epistome V–shaped, posterior angle slightly less than 90°, posteriorly carinate; mandibular scars oval; ventral margin of scrobes visible in dorsal view from antennal insertion to base of rostrum; prementum with four setae.

*Antennae* (Fig. 7): Scapes slender, subclavate, extending to region between midpoint and posterior margin of eyes at rest, 0.85× as long as funicle; funicles with desmomere 1 1.38× longer than 2, 1 and 2 elongate-clavate, 3.06× longer than all others (compared with the shortest desmomere 5), 3 and 4 equal in length, shortly clavate, 0.63× as long as 2, 5 elongate moniliform, 0.72× as long as 4, 6 1.17× longer than 5, shortly clavate, 7 as long as 3, shortly clavate; clubs with similar pubescence throughout, elongate-oval, 0.33× as long as desmomeres, 3-segmented, basal segment 1.33× longer than segment 2, this 0.89× as long as 3, 3 with a marked annulus at midpoint.

*Pronotum*: subquadratic in dorsal outline, strongly convex; anterior margin truncate, posterior margin medially slightly produced caudad; sides strongly rounded, greatest width at midpoint, gradually narrowing towards both ends, anterior margin slightly shorter than posterior one; disc smooth, median sulcus absent; posterior margin narrowly and slightly carinate; dorsal surface with dense, small and round tubercles, each tubercle with 1 scale on top; postocular lobes absent, vibrissae blond, moderately long.

*Proventriculus* (Fig. 17): Well developed, with eight sclerotized basal plates, each plate bearing two longitudinal rows of projecting setae, brush-like, apically ending with a trapezoidal zone covered with pointed inward denticles.

*Scutellum*: Not exposed.

*Elytra* (Figs 4–5): In dorsal view elongate-ovate, anterior margin medially slightly produced and elevated as prominent flange, without humeral callus; lateral margins slightly diverging from base to basal 1/5, there widest apart, then evenly converging towards narrowly rounded apex; in lateral view with posterior declivity straight and not overhanging elytral apex; with ten complete, distinct, narrow, moderately deep, punctate striae; punctures minute, moderately dense, intervals between punctures narrow; interstriae wide, slightly eminent.

**Figures 10–23.**
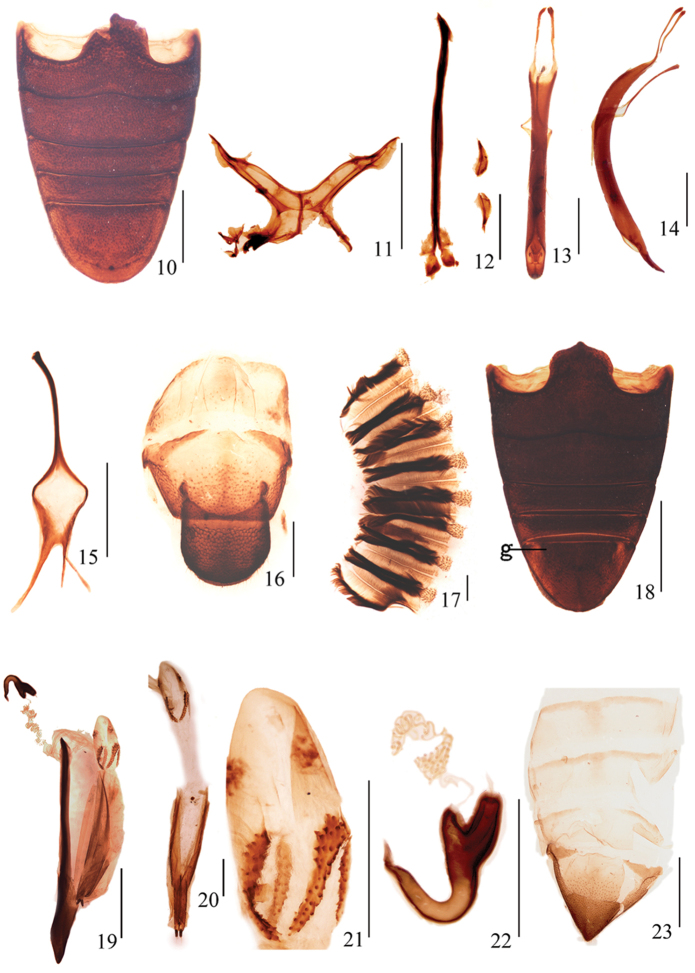
Abdominal and genital structures of *Leptomias verticalis* sp. n.: **10** male paratype, ventrites, ventral view **11** male paratype, metendosternite, dorsal view **12** male paratype, sternites VIII and IX, dorsal view **13** male paratype, aedeagus, dorsal view **14** male paratype, aedeagus, lateral view **15** male paratype, tegmen, dorsal view **16** male paratype, pygidium, dorsal view **17** male paratype, proventriculus **18** female paratype, ventrites, ventral view (g - laterobasal groove) **19** female paratype, sternite VIII and genitalia, lateral view **20** female paratype, gonocoxites and styli, dorsal view **21** female paratype, bursal sclerites, dorsolateral view **22** female paratype, spermatheca, lateral view **23** female paratype, pygidium, dorsal view. Scale bars **10–19, 23**: 1 mm; **20–22**: 0.5 mm.

*Abdomen* (Fig. 10): Ventrite 1 depressed in middle, slightly convex at sides, in middle longer than 2, suture between 1 and 2 slightly curved forward in middle,; ventrite 2 moderately convex, as long as 3 and 4 combined; ventrite 5 as long as 1 in middle, moderately convex, broadly rounded at apex.

*Metendosternite* (Fig. 11): Stalk 0.39× as long as furcal arms, trapezoidal and transverse, anterior part of longitudinal flange slightly shorter than posterior part; hemiductus inconspicuous; anterior tendons positioned almost at apical 1/3 of furcal arms; furcal arms robust, apically not bifurcate, diverging at nearly 60°in relation to longitudinal flange.

*Legs*: Procoxae situated close to front margin of prothorax; contiguous, inner side flat. Protibiae (Fig. 8) subcylindrical, slightly sinuate, bent inwards at apical 1/3; inner margin from basal 1/5 to apex arcuate, with 10 large, slightly curved, sharp teeth, each tooth with spiniform seta just behind it; Mesotibiae similar but teeth slightly smaller; metatibiae with inner margin adentate, apical 1/3 with much denser and longer setae. Tarsi slender, tarsomere 1 nearly 2× longer than 2, much wider than 2, 3 wider than the others, deeply bilobed, 5 slender; claws connate in basal half.

*Genitalia and terminalia*: Sternite VIII (Fig. 12) divided into 2 transversely orientated, crescent-shaped hemisternites, each laterally acuminate, with anterior margin moderately sinuate and posterior margin arcuate. Sternite IX (Fig. 12) with basal plate bilobed, each lobe with inner margin almost straight and outer margin Σ–shaped; spiculum gastrale 0.90× as long as aedeagus, almost straight, strongly sclerotized, anterior 1/5 slightly curved. Penis (Figs 13, 14) in dorsal view 3.90 mm long and 0.50 mm wide, temones 1.15 mm long; lateral margins subparallel in middle, minimally expanded at ostium level, thereafter slightly rounded and triangularly converging, though very narrowly rounded apex; in lateral view curved, caudally of ostium strongly constricted towards apex. Tegmen (Fig. 15) 0.46× as long as penis, ring narrow, parameroid lobes more sclerotized in basal half, separated from each other; tegminal apodeme slender, more sclerotized, Y–shaped with basal piece, slightly curved at apex.

#### Variation.

**Male paratype.** Measurements (in mm): Standard length: 8.20; pronotal length: 3.20; pronotal width: 3.20; elytral length: 5.00; elytral width: 3.30; rostral length: 1.35; rostral width: 1.20; inner margin of protibiae with eleven blunt teeth (apex worn out), mesotibiae with ten small sharp teeth.

**Female paratypes**. Measurements (in mm): Standard length: 10.40–11.30; pronotal length: 3.00–3.20; pronotal width: 3.10–3.20; elytral length: 6.00–6.70; elytral width: 3.70–3.90; rostral length: 1.29–1.40; rostral width: 1.28–1.32. Pronotum with anterior and posterior margins not truncate, slightly curved; greatest width just behind midpoint. Elytra much longer and wider than in male; in lateral view with posterior declivity straight and overhanging elytral apex; ventrite 5 (Fig. 18) parabolic, apical 1/2 with median longitudinal ridge, slightly elevated, with basal longitudinal groove on each side close to lateral margins, extending from base to midpoint (Fig. 18, g). Inner margin of pro- and mesotibiae with 8–10 larger and sharper teeth than in male, inner margin of metatibiae with 10 small and sharp teeth. Sternite VIII (Fig. 19) with spiculum ventrale clavate, wide and straight; lamina tightly folded, in lateral view triangular, strongly sclerotized, ventral margin angular in middle. Ovipositor (Figs 19, 20) with gonocoxites shorter than sternite VIII, folded along middle, lateral margins strongly sclerotized, styli relatively small, cylindrical, with 2–3 long setae inserted apically, more strongly sclerotized than gonocoxites. Bursal sclerites (Fig. 21) near point of union of spermathecal duct and oviduct, with 1 V–shaped sclerite and 1 small sclerite on each side, dorsal surface of sclerites with small and sharp conical spines. Spermatheca (Fig. 22) with corpus short, trapezoidal; cornu elongate, strongly curved into a narrow U–shape, apically gradually narrowed, apex sinuate and acuminate; nodulus prominent, tube-like, apically gradually narrowed, subcontiguous with and angled at about 60°in relation to ramus; ramus prominent, elongate trapezoid, 2.0× length of nodulus.

#### Material examined.

Holotype, ♂: (white, printed): 四川九龙县南 [Sìchuān Jǐulóngxiàn nán] J08 / 2120m 核桃林 [Hétáolín] 杯诱 [bēiyòu] / 2001.VII.9–12 于晓东 [Yú Xiǎodōng] / 中科院动物所 [Zhōngkēyuàn Dòngwùsuǒ, printed]; (white, printed): IOZ(E) 1965001. Paratypes (1♂, 4♀): 1 ♂: (white, printed): same data as holotype except IOZ(E) 1965002; 2 ♀: (white, printed): same data as holotype except IOZ(E) 1965003, IOZ(E) 1965004; 2 ♀: (white, printed): same data as holotype except J09 / 2200m 青冈灌丛 [Qīnggāng Guàncóng] and IOZ(E) 1965005, IOZ(E) 1965006.

#### Remarks.

The specific epithet refers to the straight declivity of the elytra in lateral view.

#### Distribution.

Sichuan (China).

### Other species of *Leptomias* from Sichuan

#### Leptomias elongitus

Taxon classificationAnimaliaColeopteraCurculionidae

Chao, 1981

Leptomias elongitus Chao, 1981. Insects of Xizang 1: 543, pl. III–19.

##### Type material examined.

Holotype, ♂: (white): 西藏 [Xīzàng, printed] 芒康 [Mángkāng, handwritten] / 3800 公尺 [Gōngchǐ, handwritten] / 中国科学院 [Zhōngguó Kēxuéyuàn, printed]; (white): 1976.VI.9 [handwritten] / 采集者: 韩寅恒 [Cǎijízhě, Hán Yínhéng, printed]; (red, printed): HOLOTYPE; (white, printed): IOZ(E) 906241. Paratypes: 1 ♀: same data as holotype except ALLOTYPE printed on sea-green paper and IOZ(E) 906242. 1 ♀: same data as holotype except PARATYPE printed on yellow paper and IOZ(E) 906243. 4 ♀, 2 ♂: same data as holotype except locality 芒康盐井 [Mángkāng Yánjǐng, handwritten], 2700 公尺 [Gōngchǐ, handwritten], collecting date 1976.VI.3, PARATYPE printed on yellow paper and IOZ(E) 906244–IOZ(E) 906249. 1 ♀: (white): 西藏芒康什草 [Xīzàng Mángkāng Shícǎo, handwritten] / 19 [printed] 76 [handwritten] 年 [nián, printed] 7 [handwritten] 月 [yuè, printed] 15 [handwritten] 日 [rì, printed] / 采集者 [Cǎijízhě, printed] 2600 米 [mǐ, handwritten] / 中国科学院 [Zhōngguó Kēxuéyuàn, printed]; (yellow, printed): PARATYPE; (white, printed): IOZ(E) 906250. 2♀: ditto, IOZ(E) 906251, IOZ(E) 906252.

##### Additional material examined.

1 ♂: (white, handwritten): 3 / 得荣鱼根 [Déróng Yúgēn] / 高山新 [Gāoshānxīn] / 3700 公尺 [Gōngchǐ], 何多吉 [Hé Duōjí] 80.6.8; (white, printed): IOZ(E) 906253. 1♀: ditto, IOZ(E) 906254.

##### Remarks.

**Figure 24.**
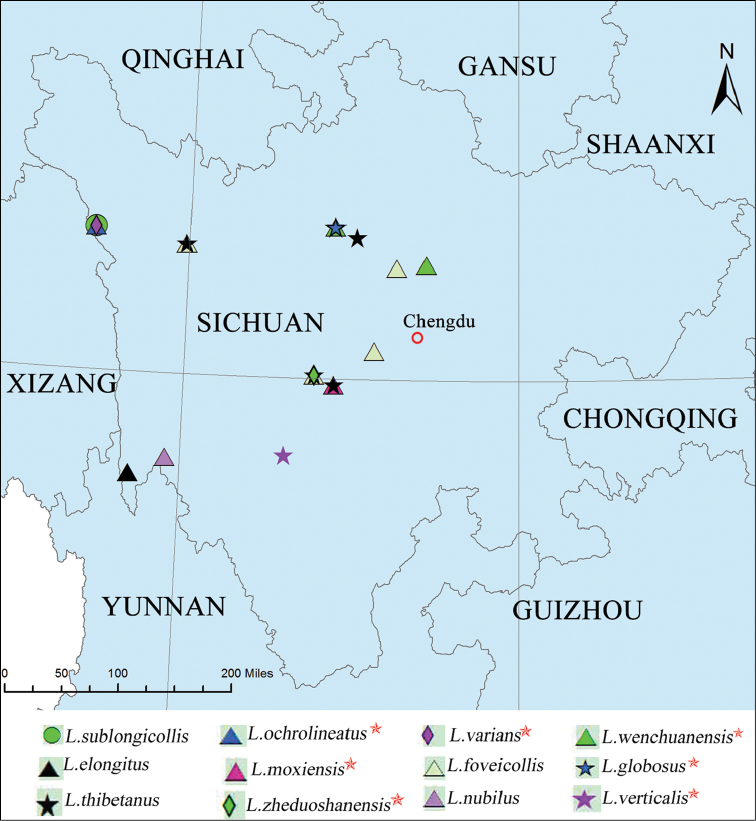
Distribution map of *Leptomias* species from Sichuan (all endemic species are marked by five-pointed stars).

*Leptomias elongitus* is known from the province of Sichuan (Derong) and Xizang (Mangkang). It is narrowly distributed in the southwest of Sichuan (Fig. 24). *Leptomias elongitus* resembles *L. nubilus* but differs by the following characters: antennae with scape extending beyond anterior margin of eye but not reaching middle when at rest; postocular lobes developed; prothorax broadest behind middle.

#### Leptomias foveicollis

Taxon classificationAnimaliaColeopteraCurculionidae

Voss, 1935

Leptomias foveicollis Voss, 1935. Entomologisches Nachrichtenblatt 9(2): 58.

##### Additional material examined.

1 ♂: (white): 四川 [Sìchuān, printed] 康定瓦新 [Kāngdìng Wǎxīn, handwritten] / 沟 [Gōu, handwritten] 1450m [handwritten] / 中国科学院 [Zhōngguó Kēxuéyuàn, printed]; (white): 1983.VI–22 [handwritten] / 采集者 王書永[Cǎijízhě, Wáng Shūyǒng, printed]; (white, printed): IOZ(E) 906879. 1 ♂: (white): 四川 [Sìchuān, printed] 康定 [Kāngdìng, handwritten] / 2600m [handwritten] / 中国科学院 [Zhōngguó Kēxuéyuàn, printed]; (white): 1983.V–30 [handwritten] / 采集者 王書永 [Cǎijízhě, Wáng Shūyǒng, printed]; (white, printed): IOZ(E) 906854. 1 ♂: same data as 906854 except collecting date 1983.VI–25, 采集者: 陈元清 [Cǎijízhě, Chén Yuánqīng, printed] and IOZ(E) 906860. 1 ♀: (white): 四川宝兴 [Sìchuān Bǎoxīng, printed] / 硗碛 [qiāoqì, printed] 2200–2700m [printed] / 1963.VI. [printed] 25 [handwritten] / 天津自然博物馆 [Tiānjīn Zìránbówùguǎn, printed]; (white): 采集者: 熊江 [Cǎijízhě, Xióngjiāng, printed]; (white, printed): IOZ(E) 906836. 2 ♀: ditto, IOZ(E) 906837, IOZ(E) 906830.

##### Remarks.

*Leptomias foveicollis* is widely distributed in Sichuan (Baoxing, Kangding, Ganzi, Lixian), Liaoning (Changtu) and Heilongjiang (Haerbing). From southwest to northeast of China, this species has a wide distribution range. *Leptomias foveicollis* is widely distributed in the central-western region of Sichuan (Fig. 24). This species is similar to *L. moxiensis* but can be differed from the following characters: prothorax strongly convex, broadest behind middle, with fovea on either side of median longitudinal groove; elytra every interstriae with 1–2 column setae; procoxae contiguous, inner sides flat; penis long and robust, apex elongate and obliquely truncate. We could not locate type materials, all above are based on identified specimens.

#### Leptomias globosus

Taxon classificationAnimaliaColeopteraCurculionidae

Chen, 1987

Leptomias globosus Chen, 1987. Acta Zootaxonomica Sinica, 12(4): 409, fig. 7.

##### Type material examined.

Holotype, ♂: (white): 四川 [Sìchuān, printed] 马尔康 [Mǎěrkāng, handwritten] / 2500m [handwritten] / 中国科学院 [Zhōngguó Kēxuéyuàn, printed]; (white): 1983.VIII.17 [handwritten] / 采集者 王書永 [Cǎijízhě, Wáng Shūyǒng, printed]; (red, printed): HOLOTYPE; (white, printed): IOZ(E) 905588.

##### Remarks.

*Leptomias globosus* is an endemic species of China which recorded from Maerkang, central region of Sichuan (Fig. 24). *Leptomias globosus* can be distinguished from other species by the following characters: disc of pronotum is strongly convex; prothorax broadest in middle; scrobes with dorsal margin not carinate; elytra posterior margin carinate, striae narrow, punctures small, every interstriae with 1–2 column setae, odd interstriae slightly raised than even ones; penis short and robust, apex short and truncate.

#### Leptomias moxiensis

Taxon classificationAnimaliaColeopteraCurculionidae

Chen, 1992

Leptomias moxiensis Chen, 1992. In Chen S (Ed) Insects of the Hengduan Mountains Region 2: 843, fig. 10.

##### Type material examined.

Holotype, ♂: (white): 四川 [Sìchuān, printed] 泸定磨西 [Lúdìng Móxī, handwritten] / 1650m [handwritten] / 中国科学院 [Zhōngguó Kēxuéyuàn, printed]; (white): 1983.VI.20 [handwritten] / 采集者: 陈元清 [Cǎijízhě, Chén Yuánqīng, printed]; (red, printed): HOLOTYPE; (white, printed): IOZ(E) 905925. Paratypes: 1 ♀: same data as holotype except ALLOTYPE printed on sea-green paper and IOZ(E) 905926. 5 ♂, 3 ♀: same data as holotype except PARATYPE printed on yellow paper and IOZ(E) 905927–905934. 1 ♂: (white): 四川 [Sìchuān, printed] 泸定磨西海 [Lúdìng Móxī, Hǎi handwritten] / 螺沟 1550m [Luógōu, handwritten] / 中国科学院 [Zhōngguó Kēxuéyuàn, printed]; (white): 1982.IX.16 [handwritten] / 采集者 王書永 [Cǎijízhě, Wáng Shūyǒng, printed]; (yellow, printed): PARATYPE; (white, printed): IOZ(E) 905935. 1 ♀: ditto, IOZ(E) 905936. 1 ♀: same data as holotype except PARATYPE printed on yellow paper, 1500m, 采集者: 张学忠 [Cǎijízhě, Zhāng Xuézhōng, printed] and IOZ(E) 905937. 1 ♂: ditto, IOZ(E) 905938.

##### Remarks.

*Leptomias moxiensis* is also an endemic species of China. It is recorded only from Luding, the central region of Sichuan (Fig. 24). *Leptomias moxiensis* can be identified by the following characters: prothorax broadest in middle, pronotum corrugated, with very fine, deep median longitudinal groove; antennae with scape reaching posterior margin of eye when at rest; elytra every interstriae with one column setae; prementum with two setae; procoxae separated from each other, inner sides flat; metatibiae with mucro.

#### Leptomias nubilus

Taxon classificationAnimaliaColeopteraCurculionidae

Chen, 1983

Leptomias nubilus Chen, 1983. Acta Zootaxonomica Sinica 8(4): 397–398, fig. 2.

##### Type material examined.

Holotype, ♂: (white): 西藏芒康 [Xīzàng Mángkāng, handwritten] / 中国科学院 [Zhōngguó Kēxuéyuàn, printed]; (white): 1977.9.15 [handwritten] / 采集者 [Cǎijízhě, printed] 李继均 [LǐJìjūn, handwritten]; (red, printed): HOLOTYPE; (white, printed): IOZ(E) 906290. Paratypes: 1 ♀: same data as holotype except PARATYPE printed on yellow paper and IOZ(E) 906291.

##### Additional material examined.

1 ♂: (white): 四川 [Sìchuān, printed] 乡城 [Xiāngchéng, handwritten] / 2900m [handwritten] / 中国科学院 [Zhōngguó Kēxuéyuàn, printed, printed]; (white): 1983.VI.28 [handwritten] / 采集者: 张学忠 [Cǎijízhě, Zhāng Xuézhōng, printed]; (white, printed): IOZ(E) 906309. 2 ♂: ditto, IOZ(E) 906310, IOZ(E) 906311. 1 ♀: same data as 906309 except collecting date 1982.VI.17, 采集者: 王書永 [Cǎijízhě, Wáng Shūyǒng, printed] and IOZ(E) 906293. 2 ♀: ditto, IOZ(E) 906294, IOZ(E) 906296.

##### Remarks.

*Leptomias nubilus* is recorded from Sichuan (Xiangcheng) and Xizang (Mangkang). Xiangcheng is located in the southwest region of Sichuan (Fig. 24). *Leptomias nubilus* can be distinguished from *L. elongitus* by antennae with scape reaching middle of eye when at rest; eyes large and slightly flat; postocular lobes not developed; with a depression positioned laterally between the upper margin of antennal scrobe and the front of eyes; prothorax rather smooth, without median longitudinal, broadest before middle.

#### Leptomias ochrolineatus

Taxon classificationAnimaliaColeopteraCurculionidae

Chen, 1987

Leptomias ochrolineatus Chen, 1987. Acta Zootaxonomica Sinica 12(4): 406–407, fig. 4.

##### Type material examined.

Holotype, ♂: (white): 四川 [Sìchuān, printed] 德格 [Dégé, handwritten] / 3200m [handwritten] / 中国科学院 [Zhōngguó Kēxuéyuàn, printed]; (white): 1983.VII.6 [handwritten] / 采集者: 陈元清 [Cǎijízhě, Chén Yuánqīng, printed]; (red, printed): HOLOTYPE; (white, printed): IOZ(E) 905520. Paratypes: 1 ♀: same data as holotype except ALLOTYPE printed on sea-green paper and IOZ(E) 905521. 8 ♀, 4 ♂: same data as holotype except PARATYPE printed on yellow paper and IOZ(E) 905522–905528, IOZ(E) 905531, IOZ(E) 905537, IOZ(E) 905540–905542. 3 ♀, 6 ♂: same data as holotype except PARATYPE printed on yellow paper, collecting date 1983.VII.4 and IOZ(E) 905529, IOZ(E) 905530, IOZ(E) 905532–905536, IOZ(E) 905538, IOZ(E) 905539.

##### Remarks.

*Leptomias ochrolineatus* is endemic to China and collected only from Sichuan (Dege). It is narrowly distributed in the northwest region of Sichuan (Fig. 24). *Leptomias sublongicollis* and *L. varians* are also recorded from the same locality, Dege; however, *L. ochrolineatus* is very different from *L. varians*: elytra in lateral view moderately flat, intervals convex, with odd intervals more raised than even ones; prothorax broadest in middle; pronotum without median longitudinal groove; penis bent downwards at apex. *L. ochrolineatus* is similar to *L. verticalis* except the following characters: prementum with two setae; antennae with scape extending beyond middle of eye when at rest; elytra in lateral view with posterior declivity not straight; scutellum large, ligulate.

#### Leptomias sublongicollis

Taxon classificationAnimaliaColeopteraCurculionidae

Chen, 1987

Leptomias sublongicollis Chen, 1987. Acta Zootaxonomica Sinica 12 (4): 404–405, fig. 1.

##### Type material examined.

Holotype, ♂: (white): 云南 [Yúnnán, printed] 维西攀天 [Wéixī Pāntiān, handwritten] / 阁 [Gé, handwritten] 2500m [handwritten] / 中国科学院 [Zhōngguó Kēxuéyuàn, printed]; (white): 1981.VII.24 [handwritten] / 采集者: [Cǎijízhě, printed] 廖素柏 [Liào Sùbó, handwritten] / (red, printed): HOLOTYPE; (white, printed): IOZ(E) 905433. Paratypes: 1 ♀: same data as holotype except ALLOTYPE printed on sea-green paper and IOZ(E) 905434. 14 ♂, 5 ♀: same data as holotype except PARATYPE printed on yellow paper and IOZ(E) 905435–905437, IOZ(E) 905448, IOZ(E) 905473, IOZ(E) 905482, IOZ(E) 905483, IOZ(E) 905485, IOZ(E) 905488, IOZ(E) 905489, IOZ(E) 905491–905493, IOZ(E) 905495, IOZ(E) 905497–905500, IOZ(E) 905503. 7 ♂, 4 ♀: same data as holotype except PARATYPE printed on yellow paper, (white, printed) 采集者 张学忠 [Cǎijízhě, Zhāng Xuézhōng] and IOZ(E) 905438, IOZ(E) 905442, IOZ(E) 905447, IOZ(E) 905452, IOZ(E) 905472, IOZ(E) 905477, IOZ(E) 905478, IOZ(E) 905481, IOZ(E) 905494, IOZ(E) 905501, IOZ(E) 905502. 12 ♂, 5 ♀: same data as holotype except PARATYPE printed on yellow paper, collecting date 1981.VII.26, IOZ(E) 905439–905441, IOZ(E) 905443, IOZ(E) 905446, IOZ(E) 905449, IOZ(E) 905454, IOZ(E) 905458, IOZ(E) 905462, IOZ(E) 905463, IOZ(E) 905465–905468, IOZ(E) 905471, IOZ(E) 905474, IOZ(E) 905496 and with 寄主: 黑桃 [Jìzhǔ, Hēitáo, handwritten]. 1 ♂, 2 ♀: same data as holotype except PARATYPE printed on yellow paper, collecting date 1981.VII.26, IOZ(E) 905444, IOZ(E) 905445, IOZ(E) 905461 and with (white, printed) 采集者 王書永 [Cǎijízhě, Wáng Shūyǒng]. 4 ♂, 4 ♀: same data as holotype except PARATYPE printed on yellow paper, collecting date 1981.VII.28 and IOZ(E) 905450, IOZ(E) 905456, IOZ(E) 905459, IOZ(E) 905460, IOZ(E) 905464, IOZ(E) 905475, IOZ(E) 905479, IOZ(E) 905484. 1 ♂, 2 ♀: same data as holotype except PARATYPE printed on yellow paper, collecting date 1981.VII.27 and IOZ(E) 905451, IOZ(E) 905469, IOZ(E) 905470. 1 ♂, 1♀: same data as holotype except PARATYPE printed on yellow paper, collecting date 1981.VII.27, IOZ(E) 905453, IOZ(E) 905455 and with (white, printed) 采集者 王書永 [Cǎijízhě, Wáng Shūyǒng]. 1 ♂, 1 ♀: same data as 905455 except collecting date 1981.VII.28 and IOZ(E) 905457, IOZ(E) 905480. 1 ♂, 1 ♀: same data as holotype except PARATYPE printed on yellow paper, collecting date 1981.VII.27, IOZ(E) 905476, IOZ(E) 905487 and with (white, printed) 采集者 张学忠 [Cǎijízhě, Zhāng Xuézhōng]. 1 ♂, 1 ♀: same data as holotype except PARATYPE printed on yellow paper, IOZ(E) 905486, IOZ(E) 905490 and with (white, printed) 采集者 王書永 [Cǎijízhě, Wáng Shūyǒng]. 1 ♀: (white): 云南 [Yúnnán, printed] 维西白济汎 [Wéixī Báijìfān, handwritten] / 1780m [handwritten] / 中国科学院 [Zhōngguó Kēxuéyuàn, printed]; (white): 1981.VII.12 [handwritten] / 采集者: [Cǎijízhě, printed] 廖素柏 [Liào Sùbó, handwritten] / (yellow, printed): PARATYPE; (white, printed): IOZ(E) 905504. 1 ♀: (white): 云南 [Yúnnán, printed] 维西白济汎 [Wéixī Báijìfān, handwritten] / 1780m [handwritten] / 中国科学院 [Zhōngguó Kēxuéyuàn, printed]; (white): 1981.VII.10 [handwritten] / 19号 [Hào, handwritten] / 采集者 王書永 [Cǎijízhě, Wáng Shūyǒng, printed] / (yellow, printed): PARATYPE; (white, printed): IOZ(E) 905505. 1 ♀: (white): 四川 [Sìchuān, printed] 德格 [Dégé, handwritten] / 3200m [handwritten] / 中国科学院 [Zhōngguó Kēxuéyuàn, printed]; 1983.VII.4 [handwritten] / 采集者: 陈元清 [Cǎijízhě, Chén Yuánqīng, printed]; (yellow, printed): PARATYPE; (white, printed): IOZ(E) 905506.

##### Remarks.

*Leptomias sublongicollis* is recorded from Sichuan (Dege) (Fig. 24) and Yunnan (Weixi). It is distributed in the Hengduan Mountains. *L. sublongicollis* resembles *L. varians*, but they are different in the following characters: scrobes with dorsal margin carinate; eyes large and convex; postocular lobes not developed; penis bent upwards at apex; rostrum dorsal surface corrugated; protibiae apex not projecting outwards.

#### Leptomias thibetanus

Taxon classificationAnimaliaColeopteraCurculionidae

(Faust, 1888)

Heteromias thibetanus Faust, 1888. Stett. Entomol. Zeit, 49(7–9): 285–286.
Leptomias thibetanus (Faust): Marshall (1916), In: Shipley AE (Ed) The Fauna of British India, including Ceylon and Burma, pp 172.

##### Type material examined.

1 ♂: Thibet / Oayrollv (white, handwritten); Coll J. Faust / Ankauf 1900 (yellow, printed); Type (red, printed); Staatl. Museum für / Tierkunde Dresden (white, printed).

##### Additional material examined.

1 ♀: (white): 四川 [Sìchuān, printed] 贡嘎山 [Gònggāshān, handwritten] / 燕子沟 2500m [Yànzǐgōu, handwritten] / 中国科学院 [Zhōngguó Kēxuéyuàn, printed]; 1983.VI.10 [handwritten] / 采集者: 陈元清 [Cǎijízhě, Chén Yuánqīng, printed]; (white, printed): IOZ(E) 906817. 1 ♀: (white): 四川 [Sìchuān, printed] 红原 [Hóngyuán, handwritten] / 3500m [handwritten] / 中国科学院 [Zhōngguó Kēxuéyuàn, printed]; 1983.VIII.27 [handwritten] / 采集者: [Cǎijízhě, printed] 牛春来 [Niú Chūnlái, handwritten]; (white, printed): IOZ(E) 906819. 1 ♂: (white): 四川 [Sìchuān, printed] 贡嘎山 [Gònggāshān, handwritten] / 燕子沟 2500m [Yànzǐgōu, handwritten] / 中国科学院 [Zhōngguó Kēxuéyuàn, printed]; 1983.VI.8 [handwritten] / 采集者: 陈元清 [Cǎijízhě, Chén Yuánqīng, printed]; (white, printed): IOZ(E) 906823. 1 ♂: (white): 四川 [Sìchuān, printed] 泸定新兴 [Lúdìng Xīnxīng, handwritten] / 1900m [handwritten] / 中国科学院 [Zhōngguó Kēxuéyuàn, printed]; 1983.VI.12 [handwritten] / 采集者: 陈元清 [Cǎijízhě, Chén Yuánqīng, printed]; (white, printed): IOZ(E) 906825.

##### Remarks.

*Leptomias thibetanus* is known from Sichuan (Ganzi, Hongyuan, Kangding, Luding) and Xizang. It is widely distributed from the central to northwest region of Sichuan (Fig. 24). *L. thibetanus* is similar to *L. zheduoshanensis*, but can be recognized by the following characters: rostrum dorsal surface with a median sulcus, extending longitudinally from antennal insertion to the vertex; elytra every interstriae with 1–2 column setae; prothorax broadest behind middle, pronotum corrugated; striae with moderately large punctures; penis slender, moderately long and straight, outline gradually converging after ostium region, apex narrowly rounded projected.

#### Leptomias varians

Taxon classificationAnimaliaColeopteraCurculionidae

Chen, 1987

Leptomias varians Chen, 1987. Acta Zootaxonomica Sinica, 12(4): 405, fig. 2.

##### Type material examined.

Holotype, ♂: (white): 四川 [Sìchuān, printed] 德格 [Dégé, handwritten] / 3200m [handwritten] / 中国科学院 [Zhōngguó Kēxuéyuàn, printed]; 1983.VII.4 [handwritten] / 采集者: 陈元清 [Cǎijízhě, Chén Yuánqīng, printed]; (red, printed): HOLOTYPE; (white, printed): IOZ(E) 905414. Paratypes: 1 ♀: same data as holotype except ALLOTYPE printed on sea-green paper and IOZ(E) 905415. 6 ♂, 3 ♀: same data as holotype except PARATYPE printed on yellow paper and IOZ(E) 905416–905424.

##### Remarks.

*Leptomias varians* is an endemic species of China, collecting from Sichuan (Dege) (Fig. 24). *L. varians*, *L. ochrolineatus* and *L. sublongicollis* are all recorded from the same county. It is close to but can be differed from *L. ochrolineatus* by these characters: elytra in dorsal view broadly ovate; prothorax broadest behind middle, with very fine and shallow median longitudinal groove; elytral intervals flat; the metatibial apex with mucro; penis bent upwards at apex. *Leptomias varians* resembles *L. sublongicollis*, but can be identified by the following characters: elytra every interstriae with 1–2 column setae; scrobes with dorsal margin not carinate; postocular lobes developed; eyes large and slightly flat; penis not bent at apex.

#### Leptomias wenchuanensis

Taxon classificationAnimaliaColeopteraCurculionidae

Chen, 1992

Leptomias wenchuanensis Chen, 1992. In Chen S (Ed) Insects of the Hengduan Mountains Region 2: 843, fig. 11.

##### Type material examined.

Holotype, ♂: (white): 四川 [Sìchuān, printed] 汶川卧龙 [Wènchuān Wòlóng, handwritten] / 1920m [handwritten] / 中国科学院 [Zhōngguó Kēxuéyuàn, printed]; (white): 1983.VII.24 [handwritten] / 采集者 王書永 [Cǎijízhě, Wáng Shūyǒng, printed]; (red, printed): HOLOTYPE; (white, printed): IOZ(E) 905907. Paratypes: 1 ♀: (white): 四川 [Sìchuān, printed] 汶川 [Wènchuān, handwritten] / 卧龙 [Wòlóng, handwritten] 1920m [handwritten] / 中国科学院 [Zhōngguó Kēxuéyuàn, printed]; (white): 1983.VII.24 [handwritten] / 采集者 王書永 [Cǎijízhě, Wáng Shūyǒng, printed]; (sea-green, printed): ALLOTYPE; (white, printed): IOZ(E) 905908. 2 ♂: same data as allotype except PARATYPE printed on yellow paper and IOZ(E) 905909, IOZ(E) 905910. 3 ♀: same data as holotype except PARATYPE printed on yellow paper, collecting date 1983.VII.25, 1780m and IOZ(E) 905411, IOZ(E) 905413, IOZ(E) 905414. 1 ♂: (white): 四川 [Sìchuān, printed] 汶川 [Wènchuān, handwritten] / 卧龙 [Wòlóng, handwritten] 1780m [handwritten] / 中国科学院 [Zhōngguó Kēxuéyuàn, printed]; (white): 1983.VII.25 [handwritten] / 采集者 王書永 [Cǎijízhě, Wáng Shūyǒng, printed]; (yellow, printed): PARATYPE; (white, printed): IOZ(E) 905912. 1 ♀: ditto, IOZ(E) 905415. 1 ♂: same data as 905912 except 1800m, 采集者 张学忠 [Cǎijízhě, Zhāng Xuézhōng, printed] and IOZ(E) 905416. 1 ♀: (white): 四川 [Sìchuān, printed] 卧龙 [Wòlóng, handwritten] / 2700m [handwritten] / 中国科学院 [Zhōngguó Kēxuéyuàn, printed]; (white): 1983.VIII.9 [handwritten] / 采集者 [Cǎijízhě, printed] 牛春来 [Niú Chūnlái, handwritten]; (yellow, printed): PARATYPE; (white, printed): IOZ(E) 905917. 1 ♀: (white): 四川 [Sìchuān, printed] 汶川 [Wènchuān, handwritten] / 1300m [handwritten] / 中国科学院 [Zhōngguó Kēxuéyuàn, printed]; (white): 1983.IX.13 [handwritten] / 采集者: 张学忠 [Cǎijízhě, Zhāng Xuézhōng, printed]; (yellow, printed): PARATYPE; (white, printed): IOZ(E) 905918. 1 ♀: (white): 四川 [Sìchuān, printed] 汶川 [Wènchuān, handwritten] / 三圣沟 2500m [Sānshènggōu, handwritten] / 中国科学院 [Zhōngguó Kēxuéyuàn, printed]; (white): 1983.VIII.6 [handwritten] / 采集者: 柴怀成 [Cǎijízhě, Chái Huáichéng, printed]; (yellow, printed): PARATYPE; (white, printed): IOZ(E) 905919. 1 ♀: (white): 四川 [Sìchuān, printed] 汶川木江坪 [Wènchuān Mùjiāngpíng handwritten] / 1200m [handwritten] / 中国科学院 [Zhōngguó Kēxuéyuàn, printed]; (white): 1983.VIII.8 [handwritten] / 采集者: 柴怀成 [Cǎijízhě, Chái Huáichéng, printed]; (yellow, printed): PARATYPE; (white, printed): IOZ(E) 905920. 1 ♀: (white): 四川 [Sìchuān, printed] 汶川映 秀 [Wènchuān Yìngxìu handwritten] / 900–1000m [handwritten] / 中国科学院 [Zhōngguó Kēxuéyuàn, printed]; (white): 1983.VIII.1 [handwritten] / 采集者: 张学忠 [Cǎijízhě, Zhāng Xuézhōng, printed]; (yellow, printed): PARATYPE; (white, printed): IOZ(E) 905921. 1 ♀: (white): 1983.VIII.3 [handwritten] / 采集者 [Cǎijízhě, printed] 柴怀成 [Chái Huáichéng, handwritten]; (white): 四川 [Sìchuān, printed] 汶川 [Wènchuān, handwritten] / 映秀 [Yìngxìu, handwritten] / 900m [handwritten] / 中国科学院 [Zhōngguó Kēxuéyuàn, printed, printed]; (yellow, printed): PARATYPE; (white, printed): IOZ(E) 905922. 1 ♀: (white): 四川 [Sìchuān, printed] 马尔康 [Mǎěrkāng, handwritten] / 2900m [handwritten] / 中国科学院 [Zhōngguó Kēxuéyuàn, printed]; (white): 1983.VIII.18 [handwritten] / 采集者: [Cǎijízhě, printed] 张学忠 [Zhāng Xuézhōng, handwritten]; (yellow, printed): PARATYPE; (white, printed): IOZ(E) 905923. 1 ♀: (white): 四川 [Sìchuān, printed] 马尔康 [Mǎěrkāng, handwritten] / 梦笔山 [Mèngbǐshān, handwritten] / 4000m [handwritten] / 中国科学院 [Zhōngguó Kēxuéyuàn, printed]; (white): 1983.VIII.19 [handwritten] / 采集者: 王書永 [Cǎijízhě, Wáng Shūyǒng, printed]; (yellow, printed): PARATYPE; (white, printed): IOZ(E) 905924.

##### Remarks.

*Leptomias wenchuanensis* is an endemic species of China and recorded from Sichuan (Wenchuan, Maerkang). It is narrowly distributed in the North Central region of Sichuan (Fig. 24). *L. wenchuanensis* and *L. globosus* are both distributed in Maerkang County, north central Sichuan. *L. wenchuanensis* is different from *L. globosus* by the following characters: protibiae strongly bent inwards at apical; inner margin of pro-, meso- and metatibiae with large teeth; prementum with two setae; pronotum strongly convex, with two foveae on either side of median longitudinal groove.

#### Leptomias zheduoshanensis

Taxon classificationAnimaliaColeopteraCurculionidae

Chen, 1992

Leptomias zheduoshanensis Chen, 1992. In: Chen S (Ed) Insects of the Hengduan Mountains region 2: 842–843.

##### Type material examined.

Holotype, ♂: (white): 四川 [Sìchuān, printed] 康定 [Kāngdìng, handwritten] / 4200m [handwritten] / 中国科学院 [Zhōngguó Kēxuéyuàn, printed]; (white): 1983.VII.13 [handwritten] / 采集者: 陈元清 [Cǎijízhě, Chén Yuánqīng , printed]; (red, printed): HOLOTYPE; (white, printed): IOZ(E) 905953. Paratypes: 1 ♀: same data as holotype except ALLOTYPE printed on sea-green paper and IOZ(E) 905954. 2 ♂, 2 ♀: same data as holotype except PARATYPE printed on yellow paper and IOZ(E) 905955, IOZ(E) 905957, IOZ(E) 905959, IOZ(E) 905961. 1 ♂: (white): 四川 [Sìchuān, printed] 康定折多 [Kāngdìng Zhéduō, handwritten] / 山 垭口 4200m [Shān Yàkǒu, handwritten] / 中国科学院 [Zhōngguó Kēxuéyuàn, printed]; (white): 1983.VII.13 [handwritten] / 采集者 王書永 [Cǎijízhě, Wáng Shūyǒng, printed]; (yellow, printed): PARATYPE; (white, printed): IOZ(E) 905956. 1 ♂: ditto, IOZ(E) 905960. 1 ♂: (white): 四川 [Sìchuān, printed] 康定 [Kāngdìng, handwritten] / 3100m [handwritten] / 中国科学院 [Zhōngguó Kēxuéyuàn, printed]; (white): 1983.VI.24 [handwritten] / 采集者 [Cǎijízhě, printed] 陈元清 [Chén Yuánqīng, handwritten]; (yellow, printed): PARATYPE; (white, printed): IOZ(E) 905962. 1 ♀: ditto, IOZ(E) 905963. 1 ♀: (white): 四川 [Sìchuān, printed] 康定 [Kāngdìng, handwritten] / 4200m [handwritten] / 中国科学院 [Zhōngguó Kēxuéyuàn, printed]; (white): 1983.VII.13 [handwritten] / 采集者: [Cǎijízhě, printed] 牛春来 [Niú Chūnlái, handwritten]; (yellow, printed): PARATYPE; (white, printed): IOZ(E) 905958.

##### Remarks.

*Leptomias zheduoshanensis* is another endemic species of China which recorded from Sichuan (Kangding). It is narrowly distributed in the central region of Sichuan (Fig. 24). *Leptomias zheduoshanensis*, *L. foveicollis* and *L. thibetanus* are all distributed in Kangding County. *Leptomias zheduoshanensis* differs from *L. thibetanus* by the following characters: prothorax broadest in middle; striae with small punctures; rostrum dorsal surface with a narrow and deep median sulcus, not reach the vertex; elytra every interstriae with one column setae; penis bent upwards at apex.

### Key to species of *Leptomias* occurring in Sichuan

**Table:** 

1	Antennae with scape extending beyond anterior margin of eye but not reaching middle when at rest	**2**
‒	Antennae with scape reaching or extending beyond middle of eye when at rest	**6**
2	Pronotum rather corrugated; scutellum invisible; procoxae separate, inner sides flat	***L. wenchuanensis***
‒	Pronotum rather smooth, not corrugated; scutellum ligulate; procoxae contiguous, inner sides convex	**3**
3	Prementum with 4 setae; elytral striae broad with large punctures; mesotibiae without mucro; metatibiae without corbel	***L. elongitus***
‒	Prementum with 2 setae; elytral striae narrow with small punctures; mesotibiae with mucro; metatibiae with narrow corbel	**4**
4	Prothorax broadest in middle, pronotum without median longitudinal groove; penis bent downwards at apex	***L. ochrolineatus***
‒	Prothorax broadest behind middle, pronotum with very fine and shallow median longitudinal groove; penis bent upwards at apex	**5**
5	Scrobes with dorsal margin not carinate; eyes large and slightly flat; postocular lobes obvious; elytral interstriae flat	***L. varians***
‒	Scrobes with dorsal margin carinate; eyes large and convex; postocular lobes absent; elytral interstriae convex	***L. sublongicollis***
6	Scrobes with dorsal margin carinate	**7**
‒	Scrobes with dorsal margin not carinate	**10**
7	Antennae with scape reaching middle of eye when at rest; eyes large and slightly flat; prothorax broadest before middle; scutellum ligulate and covered with scales; procoxae contiguous, inner sides convex	***L. nubilus***
‒	Antennae with scape extending beyond middle of eye when at rest; eyes larger or small, convex; prothorax broadest in middle or behind it; scutellum invisible; procoxae contiguous or separated, inner sides flat	**8**
8	Prothorax broadest behind middle, pronotum with fovea on either side of median longitudinal groove; penis long and robust, apex elongate and obliquely truncate	***L. foveicollis***
‒	Prothorax broadest in middle, pronotum without fovea on either side of median longitudinal groove; penis slender, apex ogival	**9**
9	Pronotum corrugated, with very fine, deep median longitudinal groove; inner margin of metatibiae with teeth; procoxae separated from each other, inner sides flat; prementum with 2 setae	***L. moxiensis***
‒	Pronotum smooth, without median longitudinal groove; inner margin of metatibiae without teeth; procoxae contiguous, inner sides flat; prementum with 4 setae	***L. verticalis***
10	Antennae with scape reaching posterior margin of eye when at rest; pronotum without median longitudinal groove, with fovea on each side of disc; scutellum invisible; procoxae separate, inner sides flat; penis short and robust, apex short and truncate	***L. globosus***
‒	Antennae with scape extending to region between middle and posterior margin of eye when at rest; pronotum with median longitudinal groove, with fovea on either side of groove; scutellum triangular; procoxae contiguous, inner sides flat; penis short or slender, apex moderately long, ogival or round	**11**
11	Antennae with scape reaching middle of eye when at rest; prothorax broadest behind middle, pronotum corrugated; striae with moderately large punctures; penis slender, apex moderately long and straight, ogival	***L. thibetanus***
‒	Antennae with scape extending beyond middle of eye but not reaching posterior margin when at rest; prothorax broadest at midpoint, pronotum smooth; striae with small punctures; penis shorter and robust, diverging at ostium level, thereafter roundly converging, then slightly diverging again and then converging to narrowly rounded, thickened apex	***L. zheduoshanensis***

### Geotragus granulatus

Taxon classificationAnimaliaColeopteraCurculionidae

(Chao, 1980) comb. n.

Leptomias granulatus Chao, 1980. Entomotaxonomia 2(1): 29.
Leptomias chenae Alonso-Zarazaga & Ren, 2013. Catalogue of Palaearctic Coleoptera 8: 89, 396 (replacement name for secondary homonymy).

#### Comments.

The correct name for this species under *Geotragus* is *G. granulatus*, not *G. chenae*, because of Art. 59.4 of the Code: “59.4. Reinstatement of junior secondary homonyms rejected after 1960. A species-group name rejected after 1960 on grounds of secondary homonymy is to be reinstated as valid by an author who considers that the two species-group taxa in question are not congeneric, unless it is invalid for some other reason.”

#### Redescription.

Body medium-sized, black to reddish brown. Eyes lateral, oval, convex, with deep and fine circumferential stria. Rostrum 1.14× longer than wide, base narrower than frons, with slightly broad, deep median sulcus, reaching vertex. Scapes short and stout, exceeding anterior margin of eyes but not surpassing middle of eyes. Funicles with desmomere 1 elongate clavate, apical stout, 1.70× longer than desmomere 2, distinctly wider than 2. Prementum with four setae. Prothorax transverse, sides evenly rounded, broadest behind middle, pronotum with extremely shallow, fine, incomplete, median longitudinal groove. Elytral interstriae slightly elevated, unequal in width, without tubercles. Proventriculus (Fig. 30): well developed, with sclerotized basal plates, each plate brush-like, apically ending with a trapezoidal zone covered with pointed inward denticles. Metendosternite (Figs 36, 39): stalk 0.23× as long as furcal arms, anterior tendons positioned almost at apical 1/2 of furcal arms; furcal arms robust, apically not bifurcate, diverging at nearly 60°in relation to longitudinal flange. Male genitalia and terminalia: sternite VIII (Fig. 31) divided into two transversely orientated, crescent-shaped hemisternites; spiculum gastrale 0.26× as long as aedeagus, almost straight, strongly sclerotized; penis (Figs 32, 33) in dorsal view 3.36 mm long and 0.36 mm wide, aedeagal apodemes 0.69 mm long, lateral margins subparallel in middle; tegmen (Fig. 34) 0.31× as long as penis, ring narrow; endophallus (Fig. 35) strongly ossification at end. Pygidium (Figs 37, 42) in dorsal view male apex broadly rounded and female apex acuminate. Hindwing (Fig. 38): generally do not possess complete venation; oblong-ovate; gradually narrowing towards end and strongly narrowing towards base; radial field, apical, medial and anal field not apparent; at the middle part of anterior margin strongly ossified; with a setae at the end. Female sternite VIII and genitalia (Fig. 40) in lateral view coxites and styli 0.55× as long as sternum VIII. Spermatheca (Fig. 41) with corpus short, trapezoidal; cornu elongate, strongly curved into a narrow U–shape, apically gradually narrowed; nodulus and ramus not developed; spermathecal duct strongly sclerotized and curved.

**Figures 25–29.**
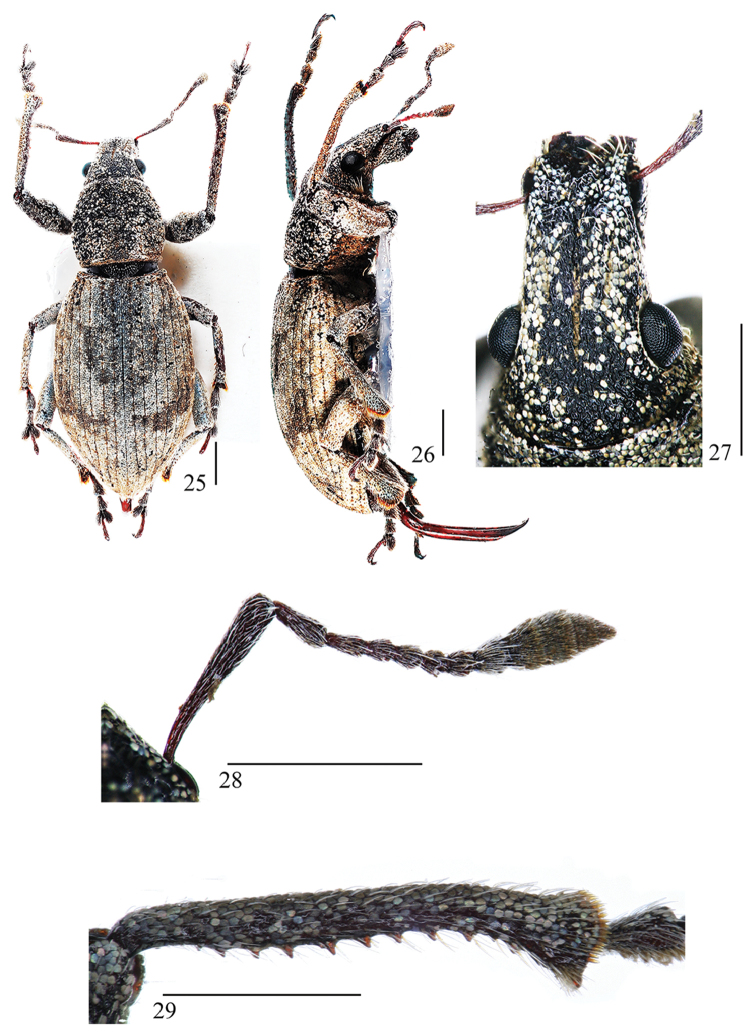
Habitus of *Geotragus granulatus* (Chao, 1980): **25** male, dorsal view **26** male, lateral view **27** male, head and rostrum, anterior view **28** male, antenna, anterior view **29** male, left protibia, anterior view. Scale bars 1 mm.

#### Remarks.

**Figures 30–42.**
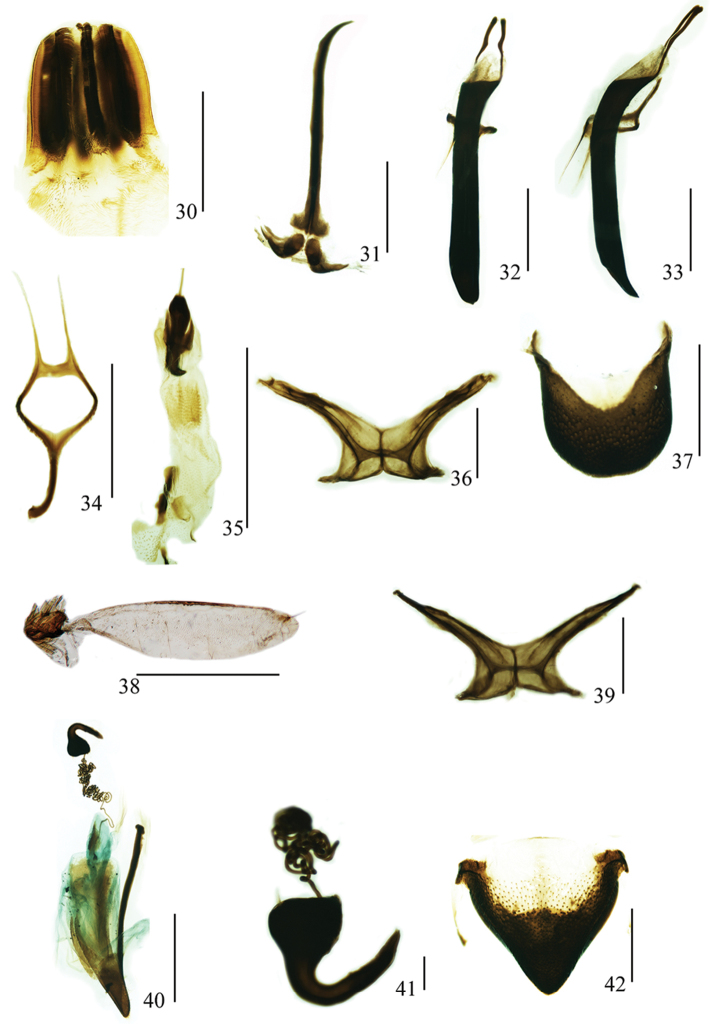
*Geotragus granulatus* (Chao, 1980): **30** male, proventriculus **31** male, sternites VIII and IX, dorsal view **32** aedeagus, dorsal view **33** aedeagus, lateral view **34** tegmen, dorsal view **35** endophallus, dorsal view **36** male, metendosternite, dorsal view **37** male, pygidium, dorsal view **38** male, hind wing, dorsal view **39** female, metendosternite, dorsal view **40** female, sternite VIII and genitalia, lateral view **41** spermatheca, lateral view **42** female, pygidium, dorsal view. Scale bars: **31–35, 37, 38, 40**: 1 mm; **30, 36, 39, 42**: 0.5 mm; **41**: 0.1 mm.

Its metanepisterna are posteriorly fused with the metaventrite and its scrobes are narrow, well defined and with the upper edge directed towards the lower end of eye, features well agreeing with the key characters of *Geotragus*. Chao (1980) described this species from a single male specimen. We later found additional specimens during several field trips, which allowed us to dissect 10 males and 10 females and illustrate the reduced hind wings and other important structures of the species (Figs 25–42). This species resembles *Geotragus declivis* Ren, Alonso-Zarazaga & Zhang, 2013 but differs by the following characters: prementum with 4 setae; elytral interstriae slightly elevated, unequal in width, without tubercles. It is similar to *Geotragus shanensis* Kumar, Mahendiran, Ayri & Ramamurthy, 2016, from which it can be distinguished by the following characters: pronotum with fine, incomplete, median longitudinal groove; protibiae only slightly bent inward at apical 1/4; bursal sclerite situated near junction of spermathecal duct and oviduct, ventral side of bursa copulatrix tile-like, in lateral view triangular, with several sharp tubercles ventrally, strongly sclerotized in middle.

#### Type material examined.

Holotype, ♂: (white, printed): 四川康定 [Sìchuān Kāngdìng] / 2400–2700 公尺 [Gōngchǐ, printed] / 中国科学院 [Zhōngguó Kēxuéyuàn]; (white): 1963.VII.28 [handwritten] / 采集者 张学忠 [Cǎijízhě Zhāng Xuézhōng, printed]; (red, printed): HOLOTYPE; (white, printed): IOZ(E) 906893.

#### Additional material examined.

8 ♂, 9 ♀: (white, printed): 四川甘孜州泸定县 [Sìchuān Gānzī Zhōu Lúdì Xiàn] / 折田坝 [Zhétiánbà] 2110m / 2011.VII.03 / 中国科学院 [Zhōngguó Kēxuéyuàn]; (white, printed): leg. 张华康 [Zhāng Huákāng] / N 29.68912 / E 102.06859 / 中国科学院 [Zhōngguó Kēxuéyuàn]; (white, printed): IOZ(E) 1506001–1506006, IOZ(E) 1506022, IOZ(E) 1506032, IOZ(E) 1506033, IOZ(E) 1506052–1506057, IOZ(E) 1506063, IOZ(E) 1506064. 1 ♂, 2 ♀: same data as 1506064 except 和平组 [Hépíngzǔ], 1845m, 2011.VI.15, N 29.64937, E 102.09164, IOZ(E) 1506027, IOZ(E) 1506031 and IOZ(E) 1506062. 3 ♂, 5 ♀: same data as 1506064 except 火草坪 [Huǒcǎopíng], 2116m, 2011.VI.30, N 29.512675, E 102.133512, IOZ(E) 1506035, IOZ(E) 1506040–1506045 and IOZ(E) 1506061. 1 ♂: (white): 四川 [Sìchuān, printed] 泸定磨西 [Lúdìng Móxī, handwritten] / 1650m [handwritten] / 中国科学院 [Zhōngguó Kēxuéyuàn, printed]; (white): 1983.VI.20 [handwritten] / 采集者: 陈元清 [Cǎijízhě, Chén Yuánqīng, printed]; (white, printed): IOZ(E) 907104. 1 ♂: same data as 907104 except 采集者 王書永 [Cǎijízhě, Wáng Shūyǒng] and IOZ(E) 907106. 1 ♀: same data as 907104 except 1500m, 采集者: [Cǎijízhě] 张学忠 [Zhāng Xuézhōng, handwritten] and IOZ(E) 907009. 1 ♀: (white): same data as 907104 except collecting date 1983.VI.17, and IOZ(E) 907010. 6 ♂, 7 ♀: same data as 907104 except 四川泸定 [Sìchuān Lúdì, printed], 1800m, 1983.VI.14 and IOZ(E) 906944–906956.

#### Distribution.

*Geotragus granulatus* mainly occurs northeast and east of Gongga Mountain, which is the highest mountain in Sichuan province, China.

## Discussion

There are 12 species of *Leptomias* occurring in Sichuan Province, accounting for 14 % of the species presently known from China. Seven of them appear to be endemic to an area that stretches from Dege County to Wenchuan County (Fig. 24). Seven endemic species are all marked on the map by five-pointed star. The other five species also occur in Xizang (Mangkang, Nielamu), Yunnan (Weixi), Liaoning (Changtu), and Heilongjiang (Haerbin) provinces. The 12 species in the centre are more widely distributed than those in the south and along the western border. Dege and Kangding all have three species. Ganzi, Maerkang, and Luding have two species. This places Sichuan third in terms of *Leptomias* diversity in China. The species occur in Sichuan at elevations between 900 and 4200 m, in a geographical rectangle delimited by 31°48.600'N 98°34.120'W and 31°27.600'N 103°36.600'W. *Geotragus* is recorded for the first time from Sichuan, which also presents a new northern-most record for the genus. *Geotragus* currently comprises 13 species, six of which occur in China.

## Supplementary Material

XML Treatment for Leptomias verticalis

XML Treatment for Leptomias elongitus

XML Treatment for Leptomias foveicollis

XML Treatment for Leptomias globosus

XML Treatment for Leptomias moxiensis

XML Treatment for Leptomias nubilus

XML Treatment for Leptomias ochrolineatus

XML Treatment for Leptomias sublongicollis

XML Treatment for Leptomias thibetanus

XML Treatment for Leptomias varians

XML Treatment for Leptomias wenchuanensis

XML Treatment for Leptomias zheduoshanensis

XML Treatment for Geotragus granulatus
